# FORTY YEARS OF CARING: THE STATE OF THE VOLUNTEER CAREGIVING MOVEMENT

**DOI:** 10.1093/geroni/igae098.3343

**Published:** 2024-12-31

**Authors:** Ronald Berkowsky

**Affiliations:** California State University Channel Islands, Camarillo, California, United States

## Abstract

With the expansion of the older adult population and its impacts on demands and costs of healthcare in the US, there has been a shift from relying on paid care work towards supporting informal unpaid caregiving. While there is an abundance of research that examines the impacts and value of informal caregiving provided by close family and friends, there is little that examines the impacts and value of volunteer caregiving (i.e., unpaid caregiving services provided by community members who do not have a close relationship with the care recipient). Services provided by community volunteers can provide needed assistance to community-dwelling older adults who encounter significant barriers to other care sources (e.g., cannot afford paid caregiving, lack access to family/friends, etc.). This community-based model of caregiving was first piloted in 1984 through support from the Robert Wood Johnson Foundation’s Faith in Action program and, in addition to promoting services that support older adults aging in place, is seen as an avenue to promote intergenerational collaboration, civic engagement, and well-being among community members. In this presentation, I present data collected in collaboration with the National Volunteer Caregiving Network (NVCN) from member sites to assess the current state of volunteer caregiving in the US. More specifically, I present data which elucidates the reach of volunteer caregiving, the characteristics of care recipients and volunteers, the services utilized, and the economic value of these services. I conclude with a discussion on the impacts of the volunteer caregiving movement as it enters its 40th year.

